# Core-Shell Nanofibrous Scaffold Based on Polycaprolactone-Silk Fibroin Emulsion Electrospinning for Tissue Engineering Applications

**DOI:** 10.3390/bioengineering5030068

**Published:** 2018-08-21

**Authors:** Trina Roy, Priti Prasanna Maity, Arun Prabhu Rameshbabu, Bodhisatwa Das, Athira John, Abir Dutta, Sanjoy Kumar Ghorai, Santanu Chattopadhyay, Santanu Dhara

**Affiliations:** 1 Biomaterials and Tissue Engineering Laboratory, School of Medical Science and Technology, Indian Institute of Technology Kharagpur, Kharagpur 721302, India; trinaroy87@gmail.com (T.R.); pritiprasanna@gmail.com (P.P.M.); arunprabhu@smst.iitkgp.ernet.in (A.P.R.); bodhisatwa.das.1985@gmail.com (B.D.); 2 Centre for Biopolymer Science and Technology (CBPST), CIPET, Kochi, Kerala 683501, India; athira.john14@gmail.com; 3 Advanced Technology Development Centre, Indian Institute of Technology Kharagpur, Kharagpur 721302, India; abirduttamechiitkgp@gmail.com; 4 Rubber Technology Centre, Indian Institute of Technology Kharagpur, Kharagpur 721302, India; sanjoypst07@gmail.com (S.K.G.); santanu@rtc.iitkgp.ernet.in (S.C.)

**Keywords:** emulsion, electrospinning, hydrophilicity, amphiphilic, tissue engineering

## Abstract

The vast domain of regenerative medicine comprises complex interactions between specific cells’ extracellular matrix (ECM) towards intracellular matrix formation, its secretion, and modulation of tissue as a whole. In this domain, engineering scaffold utilizing biomaterials along with cells towards formation of living tissues is of immense importance especially for bridging the existing gap of late; nanostructures are offering promising capability of mechano-biological response needed for tissue regeneration. Materials are selected for scaffold fabrication by considering both the mechanical integrity and bioactivity cues they offer. Herein, polycaprolactone (PCL) (biodegradable polyester) and ‘nature’s wonder’ biopolymer silk fibroin (SF) are explored in judicious combinations of emulsion electrospinning rather than conventional electrospinning of polymer blends. The water in oil (W/O) emulsions’ stability is found to be dependent upon the concentration of SF (aqueous phase) dispersed in the PCL solution (organic continuous phase). The spinnability of the emulsions is more dependent upon the viscosity of the solution, dominated by the molecular weight of PCL and its concentration than the conductivity. The nanofibers exhibited distinct core-shell structure with better cytocompatibility and cellular growth with the incorporation of the silk fibroin biopolymer.

## 1. Introduction

Tissue engineering amalgamates cells, engineered scaffold constructs, and biochemical or physicochemical cues for the repair of whole or partially injured tissue/organs. The role of tissue engineering holds great significance in the field of regenerative medicine sufficing the need for tissue constructs infiltrated with, either autologous or harvested cell source in case of severe tissue or organ damage [1,2]. Generally, three important components forms the core of tissue engineered constructs. These include stem or specialized cells, scaffold matrices, and the incorporation of growth factors which would promote adhesion, migration, and proliferation of cells on the scaffold surface [3,4]. Among these, the scaffold forms the most important prerequisite as it provides the anchorage between the diseased tissue/organ and inflowing body fluids, cells, and binding proteins. The choice of appropriate materials and scaffold fabrication techniques governs the requisite parameters needed for establishing optimum cell-material and cell–cell junction contacts [5]. 

Polyesters (PLA and PGA), polylactones (PCL), polyorthoesters, and polycarbonates have found widespread applications in tissue engineering owing to their strength coupled with biodegradability [6,7]. Recently, natural origin biopolymers including proteins (collagen, fibrin, silk and fibroin), polysaccharides (chitosan and alginate), and others are being explored extensively, either solely or in combination with synthetic polymers to impart bioactive cues and binding sites in tissue engineered scaffolds [8]. In the present scenario of tissue engineering, PCL, a FDA approved polyester, has gained a foothold in numerous applications due to its strength, tailorable biodegradability, noncytotoxicity, and biocompatibility [9,10,11]. However, PCL is inherently hydrophobic in nature, thus lacking bioactive recognition sites, which offers less conducive environment for cell infiltration and proliferation. To overcome this limitation, PCL has been blended with other biopolymers like chitosan [12,13], gelatin [14,15,16], collagen [17,18,19], and silk fibroin [20,21,22] for various applications of tissue engineering (including bone, skin, blood vessels, nerve, cartilage, etc.). Silk, a natural biopolymer, mainly comprises two proteins—light chain and heavy chain (1:1 ratio) linked by disulfide bond and it forms amphiphilic block copolymer with both hydrophobic and hydrophilic regions [23]. The presence of amphiphilicity renders silk with exceptional elasticity and strength. Together with its exceptional elasticity and minimal immunogenic response [24], silk fibroin (SF) has been universally accepted biopolymer in tissue engineering domain [25,26]. These very intrinsic disparities of both PCL and silk fibroin have been utilized in numerous research field of bone [27,28,29], skin, wound healing [30,31,32], tendon tissue engineering [33], vascular [34,35,36], and a myriad of other tissue engineering applications [37,38,39].

With material choices, scaffold fabrication technique also forms an integral part in determining the properties of the tissue engineered scaffolds (porosity, morphology, and roughness). Electrospinning offers facile and efficient method for fabricating nanofibrous structures which has similarity with native extra cellular matrix [40]. In recent times, apart from conventional electrospinning, focus has been shifted to co-axial and emulsion electrospinning for sustained delivery of growth factors, proteins, biomolecules, drugs, and food particles in biomedical applications, pharmaceuticals, and the food industry. In biomedical applications, the encapsulation of growth factors, proteins, and drugs are achieved in the core material of the nanofibers while the shell material prevents the burst release kinetics of the embedded particles, thus enhancing their controlled release over prolonged period of time [41]. In order to achieve optimum spinnability, co-axial electrospinning involves intricate needle and solution reservoir system and simultaneous adjustment of two or more solutions’ flow rate. Emulsion electrospinning, however offers simplistic, affordable, and cost-effective approach, wherein the emulsions’ stability is achieved normally by adding surfactants or emulsifiers [42].

In the present study, emulsion electrospinning of PCL and silk fibroin (SF) is explored using different benign solvent combinations compared to earlier report [43]. The study involves affordable strategy with cost-effective solution for core-shell structure of PCL-SF nanofiber without using co-axial electrospinning. Moreover, the effect of silk fibroin concentration upon the electrospun nanofibers was explored along with other essential parameters like emulsion compositions, viscosity, and conductivity for maneuvering core-shell formation. The core-shell nanofiber was further studied for physicochemical analysis and in vitro cell assays.

## 2. Materials and Methods

### 2.1. Materials

Polycaprolactone (PCL, M_n_ 70–90 kDa) and lithium bromide (LiBr) were purchased from Sigma Aldrich (St. Louis, MO, USA). Chloroform, sodium carbonate (anhydrous), and formic acid was obtained from Merck (Merck Specialities India Ltd., Hyderabad, India). Dialysis membrane (SnakeSkin™ 3.5K MWCO dialysis tubing) was obtained from Thermo Fisher Scientific (Waltham, MA, USA). *Bombyx mori* cocoons were procured locally from a nearby silk farm. Distilled water of ultrapure grade (Arium 611 UF, Sartorius, Germany) was used for all experiments. All the reagents mentioned above were of analytical grade.

### 2.2. Methods

#### 2.2.1. Extraction of Silk Fibroin

*Bombyx mori* cocoons were degummed and processed to obtain silk fibroin following a previous study [44] with minor modifications. Briefly, cocoons from *Bombyx mori* were boiled in an aqueous solution of 0.02 M Na_2_CO_3_ for 1 h and rinsed 4–5 times with ultrapure distilled water. The obtained degummed fibers were kept overnight for drying after which it was dissolved in lithium bromide solution at 60 °C. The fibers were dissolved to obtain a viscous solution which was dialyzed (Mw = 3.5 kDa) against ultrapure distilled water for 3 days by changing water after every 10–12 h, to remove the ions and other impurities. The dialyzed solution was collected, filtered and lyophilized to obtain silk fibroin (SF) sponges.

#### 2.2.2. Emulsion Electrospinning

PCL was dissolved in chloroform (10% *w*/*v*) and SF was dissolved in formic acid (10% and 5% *w*/*v* respectively). The solution of PCL and SF were mixed in three different ratios for PCL-SF (10%-10%) and PCL-SF (10%-5%)-70:30, 50:50, 30:70, giving a total of six different emulsions for electrospinning. All the emulsions were stirred in a magnetic stirrer (IKA) for 12–14 h, at high 1000 rpm to obtain uniform emulsion. For electrospinning, 5 mL capacity of syringe loaded with emulsion was attached to a needle tip having 0.5 mm diameter. After initial optimization, 4.0–6.0 µL/min flow rate of emulsion was maintained by syringe pump (KD Scientific, Holliston, MA, USA) and a voltage of 22–23 kV (30 kV, Glass Mann, Japan) was maintained between the needle tip and collector, kept at a distance of 10–12 cm apart for nanofiber collection. All the experiments were carried out at ambient environments. The electrospun nanofibers were collected from the collector and subjected to methanol treatment for 30 min to stabilize the silk fibroin in the electrospun mats. Thereafter, the electrospun mats were washed repeatedly with distilled water to remove any trace amount of methanol present in the scaffolds and air/oven dried for 2 days. The as-spun nanofiber mats were stored in vacuum for further studies.

#### 2.2.3. Optical Microscopy of Emulsion Droplets

The occurrence and distribution of the emulsion droplets was observed under an inverted microscope (AxioVision, Carl Zeiss, Oberkochen, Germany). The emulsion drops were taken on a glass slide and imaging was done immediately in bright field mode to capture the morphology of emulsified systems.

#### 2.2.4. Electron Microscopy of Nanofibers

The microstructure of the samples was observed under scanning electron microscope (MERLIN FESEM, Carl Zeiss, Oberkochen, Germany) at an accelerating voltage of 10–20 kV. Prior to observation, the samples were arranged on metal stubs and gold coated in vacuum for 100 s, using a plasma gold coater. The obtained fiber diameters were measured using Image software (NIH, Bethesda, MD, USA), in 50 random positions for fiber diameter distributions.

#### 2.2.5. Transmission Electron Microscopy of Core-Shell Structure

Transmission electron microscope (TEM, FEI-TECNAI G2 20S-TWIN, FEI, Hillsboro, OR, USA) was performed to ascertain the core-shell structure of nanofibers. The nanofibers with different combinations of PCL-SF were directly electrospun on the carbon coated grids of TEM and observed under 80 kV, low voltage was preferred for nanofibers to avoid radiation damage.

#### 2.2.6. FTIR Analysis

The characteristic spectral absorption peaks of PCL, PCL-SF nanofibers and SF powder were determined by FTIR spectrophotometer (NEXUS-870, Thermo Nicolet Corporation, Thermo Fisher Scientific, Waltham, MA, USA). SF powder was characterized by forming pellets with potassium bromide (KBr) and nanofiber mats were directly characterized in attenuated total reflectance (ATR-FTIR) mode with ZnSe crystal ATR accessory. All the spectra were recorded between 4000 and 500 cm^−1^. The spectral resolution was 2 cm^−1^.

#### 2.2.7. Viscosity and Conductivity 

Viscosity measurements of different PCL-SF concentration solution was carried out using Bohlin CVO Rheometer (Malvern Instrument, Malvern, UK), at different shear rates ranging from 0.1 to 500 s^−1^ at 25 °C. The spindle arrangement was parallel plate geometry with a gap of 100 µm and 30 mm diameter plate.

Along with viscosity, conductivity of different PCL-SF concentration was measured with a pH meter (Thermo Scientific, Orion VERSA STAR, Thermo Fisher Scientific, Waltham, MA, USA). Before measurement, the instrument was calibrated with solutions having conductivity of 1413.0 µS/cm and 12.9 mS/cm, respectively.

#### 2.2.8. Contact Angle 

The samples were tested for measurement of contact angles to assess the hydrophobicity/hydrophilicity of the electrospun nanofibers. Approximately a 100 μL water droplet was casted onto flat nanofibers and contact angles were measured by the sessile drop method (Rame-Hart Instrument Co., Model: 190F2, Succasunna, NJ, USA). All the images were captured after 60 s of water droplet casting.

#### 2.2.9. Swelling Study

The swelling ratio test was carried out as follows:

Dry scaffolds were weighed (W_d_) initially and then hydrated in PBS for 1 h, 3 h, 6 h, 12 h, 24 h, and 48 h, at 37 °C respectively. The excess surface water was removed with filter paper; the wet scaffolds were weighed (W_w_) again. The swelling ratio of the scaffolds was measured as the wet weight increase (W_w_−W_d_) to the initial weight (W_d_).

#### 2.2.10. Mechanical Properties

The electrospun nanofibrous sheets with 50 mm × 10 mm dimension were subjected to tensile testing [45] following some modifications. The samples (n = 5 for each sample) with gauge length 20 mm, was pulled with 100 N load cell in a UTM machine (H25KS, Hounsfield, UK) at a cross-head speed of 5 mm/min until failure. All the samples were hydrated in PBS for 3 h at 37 °C before tensile testing to simulate the hydrated environment at the human body temperature.

#### 2.2.11. In Vitro Cell Culture Studies

For in vitro cell culture studies, MG63 cells were cultured in α-MEM (Himedia) supplemented with 10% fetal bovine serum (Gibco, Invitrogen, Thermo Fisher Scientific, Waltham, MA, USA) and 1% penicillin-streptomycin antibiotic (Gibco, Invitrogen, Thermo Fisher Scientific, Waltham, MA, USA) under standard cell culture conditions of 37 °C and 5% CO_2_ in an incubator. Prior to cell seeding, the nanofiber mats (5 mm × 5 mm) were sterilized using standard methods. The mats were kept under UV sterilization for 2 h followed by 70% alcohol (ethanol/propanol) treatment for 1 h. Thereafter, the scaffolds were washed with sterile PBS thrice for 15 min. After that, the scaffolds were kept in culture media overnight. The next day the media were discarded and 1 × 10^4^ cells were seeded per scaffold supplemented with cell culture medium and incubated for 3 and 5 days. After the respective time point, the media was discarded from the culture well and the samples were washed once with PBS mildly. The cells were fixed with 4% paraformaldehyde in PBS (pH 7.4) followed by graded ethanol/propanol treatment. The fixed cells were observed under electron microscope (MERLIN FESEM, Carl Zeiss, Oberkochen, Germany) at an accelerating voltage of 10–20 kV with gold coating prior to imaging. The samples after fixation were permeabilized by standard method and stained using Rhodamine-phalloidin (R415, Thermo Fisher Scientific, Waltham, MA, USA) and DAPI (Thermo Fisher Scientific, Waltham, MA, USA) according to manufacturer’s protocol. The assessment of cell viability on samples after seeding with MG63 cells was determined using live/dead assay kit (Life Technologies, Thermo Fisher Scientific, Waltham, MA, USA) following manufacturer’s protocol. Briefly, after 3 and 5 days of cell seeding the samples were washed once with PBS and incubated with 2 μM calcein acetomethoxy (AM) and 4 μM ethidium homodimer solution for 30 min. Thereafter, the samples were washed repeatedly with PBS to avoid any nonspecific staining. All the fluorescence images were observed under an inverted fluorescence microscope for imaging (AxioVision, Carl Zeiss, Oberkochen, Germany). All the tests were performed in triplicate (n = 3) for twice.

#### 2.2.12. Statistical Analysis

Statistical analysis was performed using SPSS software by one-way ANOVA. The results obtained were expressed as a mean ± standard deviation. Significance was determined at *p* < 0.05.

## 3. Results and Discussion

### 3.1. Optical Microscopy of Emulsion Droplets

**Figure 1:**
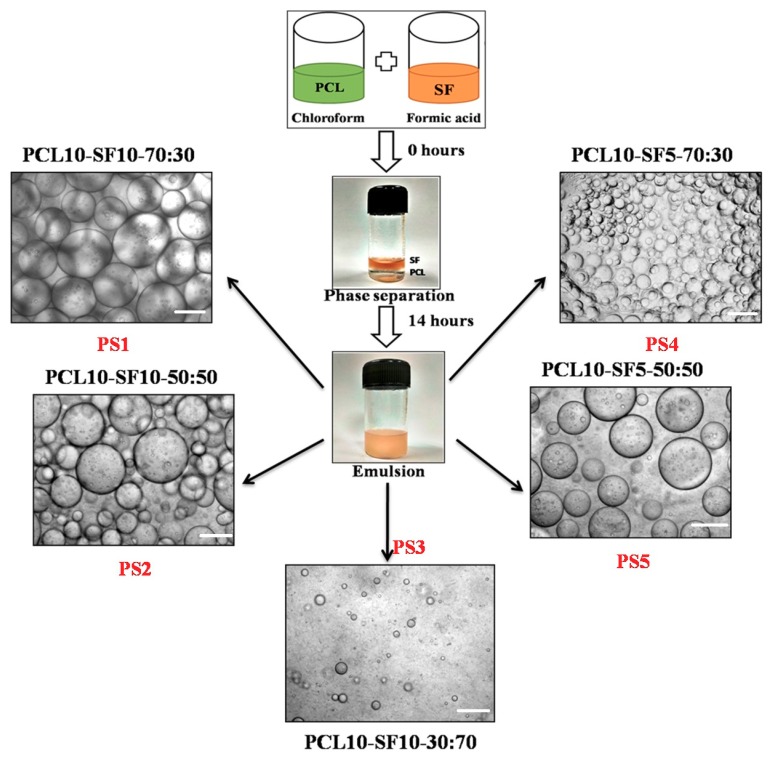
Different combinations of PCL and silk fibroin (SF) emulsion with their nomenclature, scale bar of optical images: 100 µm.

The emulsions formed after 14 h of uniform mixing at very high rpm, were observed under an inverted microscope to study the stability of the emulsion droplets (Figure 1). PCL dissolved in chloroform and silk fibroin dissolved in formic acid constituted the ‘organic phase’ and ‘aqueous phase’ in the emulsion, respectively. The concentration of PCL being kept constant at 10% (*w*/*v*), silk fibroin (SF) concentration was varied to study the effects of incorporating different silk variation upon the physicochemical, mechanical, and interfacial properties of the nanofibrous electrospun mats. 10% (*w*/*v*) and 5% (*w*/*v*) of SF was chosen as the ‘aqueous phase’ in the whole emulsion system. The different combinations of electrospinning were named accordingly: (PCL 10%-SF 0%)-PS0, (PCL 10%-SF 10%-70:30)-PS1, (PCL 10%-SF 10%-50:50)-PS2, (PCL 10%-S10%-30:70)-PS3, (PCL 10%-SF 5%-70:30)-PS4, and (PCL 10%-SF 5%-50:50)-PS5.

The polymer solution, which formed the droplets in the emulsion, comprises the ‘dispersed phase’ and the surrounding dispersing polymer solution forms the ‘continuous phase’. In our study, aqueous droplets of SF in formic acid were dispersed in the oil phase of PCL in chloroform, thus forming W/O emulsion. The emulsion droplets in PS1 and PS4 exhibited optimum uniform dispersion of SF (liquid phase) in PCL solution (organic phase) compared to other emulsion combinations. PS2 and PS5 depicted comparatively random distribution and nonuniform diameter of droplets. PS3 resulted in minimum distribution of emulsion droplets with completely erratic diameters. The emulsions formed with concentration of 10% (*w*/*v*) SF was found to have more compact arrangement of emulsified droplets than with concentration of 5% (*w*/*v*) SF. 

Furthermore, it was observed that with increasing concentration of SF in the individual emulsions, the stability of the emulsions was reducing with droplets coalescing together forming bigger droplets. This fact could be attributed to the increase in the high internal phase volume fraction of the emulsion combination [46]. SF droplets formed the dispersed phase, separated from each other by thin film of continuous phase (PCL). With increase in the concentration of SF in the PS2 and PS5 combination of emulsion, the internal phase volume fraction increased, resulting in deformation of the dispersed phase droplets leading to coalescence. It was noted that even without using any emulsifying agent or surfactant, the emulsion PS1 and PS4 was stable up to 2–3 h. However, PS2 and PS5 were stable for about an hour before phase separation occurred. PS3 emulsion exhibited unstable, random droplets arrangement with most of the droplets merging within few minutes.

### 3.2. Electron Microscopy of Electrospun Nanofibers

Electron microscopy images revealed the nanofibrous morphology of the electrospun nanofibers. High voltage connected to the metal needle reached the polymer solution forming jet instability. After overcoming the opposing surface tension in the polymer solution, the flowing polymer nanofibers experienced bending instability. With more travelling distance, the continuous phase of the emulsion evaporated resulting in an increase of viscosity. Simultaneously, the ‘dispersed phase’ in the emulsion tend to move inwards owing to the generated viscosity gradient from ‘continuous phase’ evaporation. The resulting nanofibers were deposited on the collector with core-shell structure.

**Figure 2:**
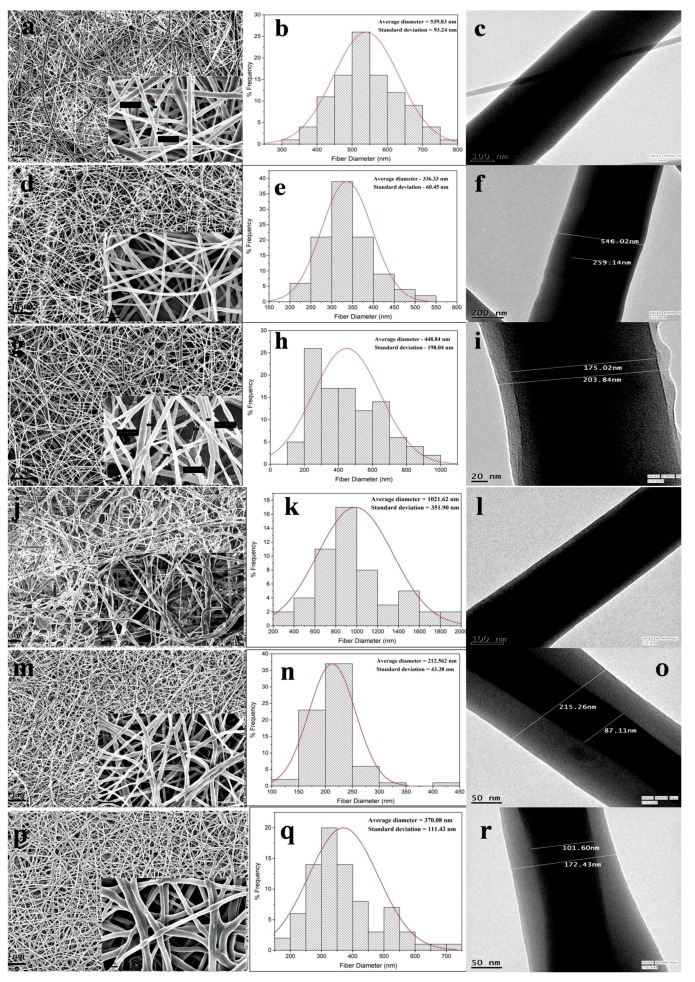
Scanning electron microscope (SEM) images of PS0 to PS5 nanofibers (**a**,**d**,**g**,**j**,**m**,**p**) (scale bar: 10 µm) with magnified images of same (inset) (scale bar: 1 µm). Frequency distribution of nanofiber diameters of PS0 to PS5 (**b**,**e**,**h**,**k**,**n**,**q**). Transmission electron microscope (TEM) images of PS0 to PS5 nanofibers (**c**,**f**,**i**,**l**,**o**,**r**).

The spinnability of emulsion combinations and nanofiber diameters are represented in Table 1. Native PCL nanofibers showed smooth fiber morphology with no beads (Figure 2a), but the diameter distribution curve of the PCL fibers depicted wide variation in the nanofiber diameter range (average diameter—539.8 ± 93.2 nm). The diameter distribution curve was obtained from the processing of scanning electron microscopy (SEM) images in Image software. PS1 nanofibers was found to have uniform, unbeaded, thin diameters (Figure 2d) with comparatively less diameter distribution variation (average diameter—336.3 ± 60.5 nm). With the incorporation of SF in the PCL organic phase, a gradient in surface tension and viscosity emerged during the flow of emulsion solution. The flow rate had to be increased compared to native PCL electrospinning; as a result fibers were drawn with more force resulting in comparatively thinner diameter and distinct fibers.

In PS2 combination, secondary, very thin fibers were produced along with nanofibers (Figure 2g). The nanofibers were bead-free but the diameter distribution was not uniform (Figure 2h) with average diameter of 448.8 ± 198.0 nm. The formation of secondary jets could be attributed due to delayed evaporation of dispersed aqueous SF phase. With increase in the SF concentration in PS2, the continuous phase might be evaporated faster than the aqueous phase due to lesser volatility of formic acid. The nanofibers in PS3 combination yielded completely random, nonuniform, beaded, and widely varying fiber diameters (Figure 2j). The increase in the SF aqueous phase affected the stability of emulsion, which ultimately yielded haphazard nanofibers (average diameter—1021.6 ± 351.9 nm). 

The nanofibers obtained from PS4 showed fine uniformity in fiber morphology (Figure 2m) with very narrow variation in fiber diameters (average diameter—212.6 ± 43.4 nm). In this combination the concentration of SF was 5% (*w*/*v*), which further yielded narrower fiber diameters compared to 10% (*w*/*v*) concentration of SF. PS5 combination also yielded nanofibers with thinner diameter but the nanofibers were more random in diameter distribution (Figure 2p,q). 

### 3.3. Transmission Electron Microscopy of Electrospun Nanofibers

Transmission electron microscopy (TEM) was performed to ascertain the core-shell structure of the electrospun nanofibers. With the initiation of the bending instability during electrospinning, the continuous phase of the polymer solution evaporates faster than the dispersed aqueous phase owing to differences in viscosity and surface tension. As the polymer jet is drawn more towards the collector plate under high electrical voltage, the aqueous phase is drawn inwards in the individual fibers giving rise to core-shell structure. 

Along with uniform fiber diameter, PS1 nanofibers exhibited distinct core-shell structure with core and shell thickness of 259.1 nm and 143.4 nm, respectively (Figure 2f). In PS2 combination, with increase in SF concentration to 50%, both the core and shell thickness decreased compared to PS1 combination, with almost merging of the core with outer shell boundary (Figure 2i). The core and shell thickness was found to be 203.8 nm and 14.4 nm, respectively. PS3 combination yielded nanofiber structure with nonconforming core-shell structure, thus the measurement was not available (Figure 2l). PS4 revealed much thicker shell layer coupled with thinner core boundary, compared to previous emulsion combination (core and shell thickness of 87.1 nm and 64.0 nm, respectively) (Figure 2o). In PS5 combination, again the thickness of the core was found to be increased with thinner layer of shell layer (Figure 2r). The core and shell thickness was found to be 101.6 nm and 35.4 nm. It could be hypothesized that, the concentration of SF played the dominant role in determining the core and shell thickness of individual nanofibers. In the PS2 combination (PCL 10%-SF 10%-50:50), when the ratio of the silk in the emulsion increased the thickness of the core also increased compared to PS1 (PCL 10%-SF 10%-70:30). Further, when the overall concentration of silk decreased from 10% to 5% in the weight ratio, the core thickness decreased, with much thinner core observed in PS4 (PCL 10%-SF 5%-70:30) than in PS5 (PCL 10%-SF 5%-50:50). 

Since, from the photomicrograph the core part of the fiber appeared to be darker, it reflected more electron density there. SF is more crystalline than PCL, hence it could be presumed that silk fibroins (SF’s) were highly internalized in the core part. Additionally, the concentration of SF in the emulsion affected the thickness of core-shell in the nanofibers. It should be noted, due to the random arrangement of nanofibers in TEM grids, all the TEM images could not be taken in the same scale.

### 3.4. FTIR Analysis

**Figure 3:**
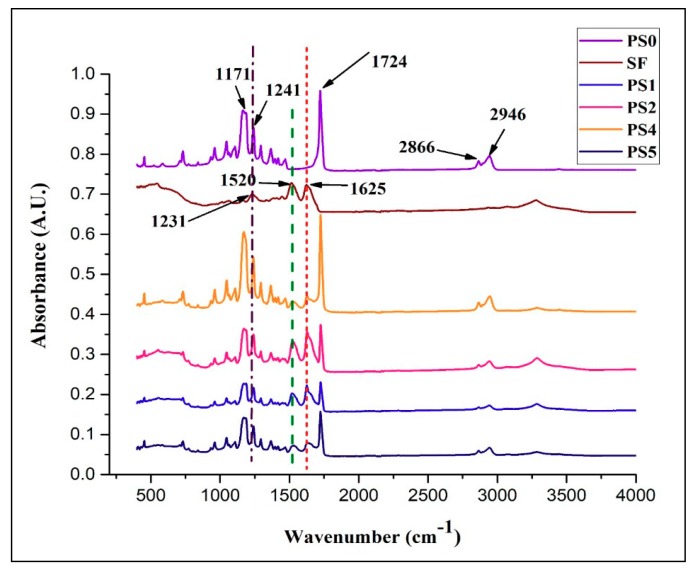
FTIR spectra of different nanofibrous scaffolds and SF powder.

The FT-IR spectra with characteristic absorption peaks of PCL, SF, and the scaffolds are presented in the Figure 3. Lyophilized SF powder was treated with methanol to impart β-sheet conformation. After evaporation of the solvent, the SF powder was dried completely before the measurement. The sharp peak at 1722 cm^−1^ denoted the C=O carbonyl ester stretching in pristine PCL nanofibers. The peak appearing at 1241 cm^−1^ represented the C–O and C–C stretching in PCL. Alongside, peaks 1180 cm^−1^ and 1171 cm^−1^ signified the asymmetric C–O–C stretching and symmetric C–O–C stretching in the PCL chains. Additionally, 2866 cm^−1^ and 2946 cm^−1^ denoted the C–H plane vibration of PCL main chains. In SF, bands 1625 cm^−1^, 1520 cm^−1^, and 1231 cm^−1^ were assigned to amide I (C=O stretching vibration), amide II (secondary N–H bending vibration) due to β-sheet structure, and amide III (complex bands of combination of C–N, N–H vibrations). Amide I and amide II formed the major bands in the silk fibroin (SF) protein structure. In the scaffolds ranging from PS1 to PS5, all the bands corresponding to PCL and SF could be identified with slight shift in the representative peaks (denoted by hashed lines), thus confirming the presence of individual vibrations in the electrospun scaffolds.

### 3.5. Measurements of Viscosity and Conductivity 

**Figure 4:**
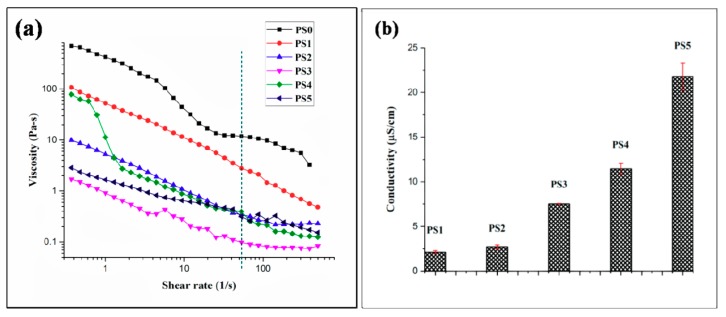
(**a**) Viscosity of different electrospinning solution (the green dashed line represent the viscosity value of emulsions at the particular shear rate 54 s^−1^) and (**b**) conductivity of different electrospinning solution.

Viscosity of the electrospinning solution plays a dominant role in determining the nanofiber formation ability, dimension and texture of the fibers. All the emulsion combinations exhibited non-Newtonian shear thinning property under the application of shear rate (Figure 4a). It has been reported [47] that sufficient chain entanglement was necessary among the polymers for initiation of uninterrupted electrospinning yielding fibers in diameter submicron range. Viscosity with both very low and high values might result in beaded nanofibers; an optimum value of viscosity in the range of 0.1 to 2 Pa-s is preferred for spinning [48]. In the following study, it was observed (Figure 4a) that PCL solution displayed higher viscosity and the stability of the nanofibers produced was intact but had thicker fiber diameters. This could be linked with high amount of polymer chain entanglement, on the contrary, with increasing SF concentration in the emulsions no significant change in viscosity was observed. This might be due to the fact that PCL comprises of major ‘continuous phase’ while SF formed minor ‘dispersed phase’. All the emulsion blends displayed spinnability except PS3 (PCL 10%-SF 10%-30:70). The viscosity of the latter was below 0.1 Pa-s which resulted in breakage of solution into droplets giving rise to multiple beaded fibers. This might be possibly the interfacial tension dominates over the viscous force in the latter case.

Conductivity of the emulsion blends was carried out at ambient environmental conditions. Reduction in the silk concentration from 10% to 5% coupled with viscosity declination resulted in elevation of conductivity values. With high viscosity of emulsion blends, the cohesion among the polymer molecules might hinder the conductivity of the solution. In this regard, it was observed with lesser conductivity values the emulsion showed appreciable spinnability (Figure 4b). Though, viscosity and conductivity of a solution is not inter-related at ambient temperature, but it could be hypothesized that with the synergistic effects of optimum high viscosity and low conductivity, better emulsion electrospinning is facilitated. All the emulsion blends displayed a specific pH range (2–2.1).

### 3.6. Contact Angle Measurement

The wettability of the biomaterials, evaluated in vitro by contact angle measurement forms the niche of fluid-material interaction, particularly adhesion between cells, proteins, and the scaffold/implant surface implanted in vivo. It has been reported that the cell adhesion and extra cellular matrix deposition are influenced by adhesion of proteins on the biomaterial scaffold surface, which in turn is governed by the surface wettability of the material itself [49].

**Figure 5:**
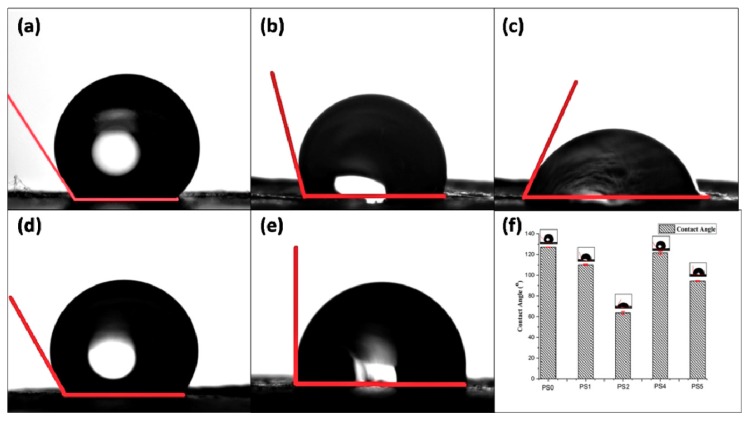
Contact angle measurement of (**a**) PS0, (**b**) PS1, (**c**) PS2, (**d**) PS4, (**e**) PS5, and (**f**) graphical representation of contact angle of nanofibers.

In the present study, the contact angle of pristine PCL nanofiber mat (PS0) was observed to be 127.0° owing to the hydrophobic nature of PCL (Figure 5a). But in the emulsion, with the addition of SF there was declination of hydrophobicity due to the presence of natural hydrophilic amino, carboxylic, and other functional groups in the backbone of SF. It was observed that elevating the silk concentration in the emulsion (50:50) rendered the nanofiber surface PS2 moderately hydrophilic (CA: 63.6°) (Figure 5c), compared to (70:30) PS1 (CA: 110.1°) (Figure 5b, respectively) [43]. Relating to the TEM images, it could be observed that the core of PS2 fibers (Figure 2i) was mostly occupied by SF with very thin layer of PCL shell (28 nm shell thickness). Decreasing the silk concentration in the emulsion from 10% to 5% *w*/*v* elevated the hydrophobicity of the scaffold surface (CA > 80°) for both PS4 and PS5 (Figure 5d,e, respectively). Again, relating to the TEM observations, it was observed that decreasing SF concentration in the emulsion, yielded thicker shell of PCL (Figure 2o,r) which might render the surface of PS4 and PS5 fibers hydrophobic. It could be predicted that cell adhesion and migration would be more pronounced on the moderately hydrophilic surface than the other nanofiber surfaces [50].

Surface roughness measurement of the electrospun scaffolds are presented in Figure S1 and tabulated in Table 2. The relationship between roughness and wettability of a surface has been described by Wenzel [51]. According to him, a chemically hydrophobic surface would become even more hydrophobic when surface roughness is added to it. Similarly, chemically modified hydrophilic surfaces would attain more hydrophilicity with incorporation of surface roughness. Herein, particularly the surface roughness of PS2 was highest (6.24 µm) followed by PS5 (5.52 µm). Both the scaffolds have higher weight percentage of SF compared to PS1 and PS4. The presence of hydrophilic functional groups in SF enhanced the wettability of PS2 and PS5 [43]. It could be hypothesized that the increase of surface roughness affected the wettability of PS2 and PS5 scaffolds.

### 3.7. Swelling Study

**Figure 6:**
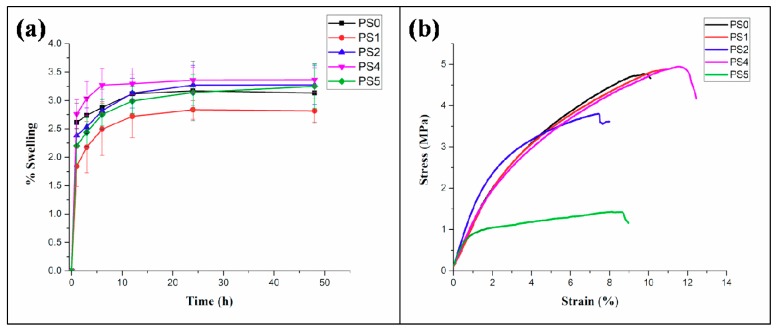
(**a**) Swelling percentage of nanofibers in PBS (pH: 7.4) at different time intervals, (**b**) tensile testing of nanofibers.

The equilibrium swelling behavior of the nanofibers was measured in PBS (pH: 7.4) at 37 °C. The stability of the nanofibers subjected to physiological environment in human body forms important criteria in determining nutrient transport, gaseous exchange, and tissue regeneration capability of the scaffolds with the native tissue environment. All the nanofibers exhibited equilibrium in swelling, particularly after 12 h with the swelling percentage found to be around ~3.3% maximum value for PS4 and ~2.5% minimum value for PS1 electrospun mats, respectively (Figure 6a). Such low percentage of swelling could be attributed to the presence of PCL, hydrophobic polyester in combination with SF, an amphiphilic biopolymer. The swelling percentage was found to be more in nanofibers with 5% SF concentration than 10%.

### 3.8. Mechanical Properties

The stress-strain curve for tensile testing of the core-shell nanofibers is presented in Figure 6b. The results indicated (Table 2) that the elastic modulus of the electrospun nanofibers were observed to increase compared to the native PCL nanofibers. A significant increase in the value of the elastic modulus was observed in the PS2 nanofibers compared to the PS1 nanofibers. A similar trend was evident in the elastic modulus values of PS5, compared to the PS4 nanofibers. The maximum strain, generated in the nanofibers was found for the PS4 nanofibers, followed by PS1, PS2, and PS5, in a descending order. Maximum ultimate tensile strength of 4.9 ± 0.18 MPa was observed for the PS4 nanofibers, whereas minimum value of the same was recorded as 1.3 ± 0.14 MPa for PS5 nanofibers. 

The increase in the elastic modulus was significant for PS4 and PS1 nanofibers, both having low weight percentage of the SF and high weight percentage of PCL in their compositions, respectively. On the contrary, PS2 and PS5 nanofibers had high SF content and low PCL content in their compositions. This may be corroborated with the fact that high SF content contributed to the stiffness of the nanofibers whereas, high PCL content enhanced the ductility property of the nanofibers resulting into high strains in the nanofibers. Moreover, it seems that SF played a crucial role to enhance the structural integrity, resulting in high elastic modulus, whereas PCL, being a thermoplastic polymer, improved the ductility of the nanofibers. 

Furthermore, the mechanical properties of electrospun sheets are significantly impacted by the orientation of nanofibers deposited. Herein, the random orientation of nanofibers might affect the mechanical property of individual fibers which influences the bulk mechanical properties. 

### 3.9. In Vitro Cell Culture Studies

**Figure 7:**
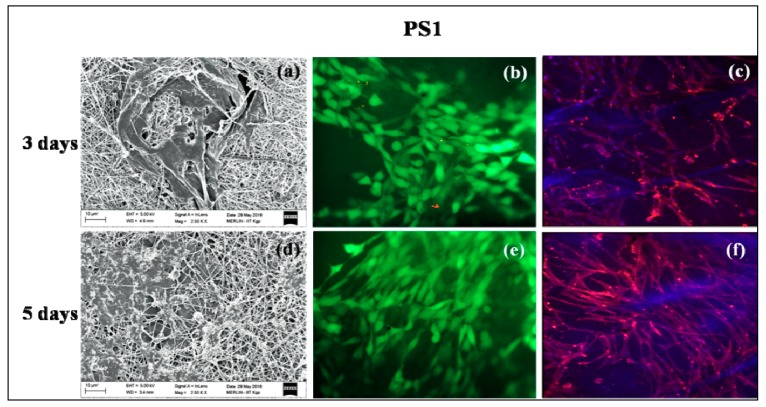
In vitro cell culture study on PS1 nanofibers: SEM (**a**) 3 days, (**d**) 5 days; Live-dead staining (**b**) 3 days, (**e**) 5 days and Rhodamine-DAPI staining (**c**) 3 days, (**f**) 5 days.

**Figure 8:**
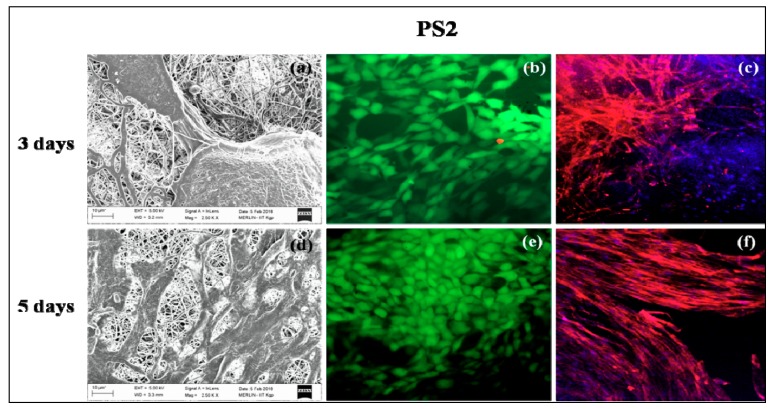
In vitro cell culture study on PS2 nanofibers: SEM (**a**) 3 days, (**d**) 5 days; Live-dead staining (**b**) 3 days, (**e**) 5 days and Rhodamine-DAPI staining (**c**) 3 days, (**f**) 5 days.

PS1 and PS2 were chosen for in vitro cell culture studies. PS1 was observed to have cell adherence in cluster forms rather than evenly distributed cell adherence and proliferation both in 3 days (Figure 7a) and 5 days study (Figure 7d). Initially, PS2 also exhibited cell cluster formation (Figure 8a) but with increasing days a monolayer of cell sheet was observed after 5 days (Figure 8d). In the live dead assay, both PS1 and PS2 exhibited cellular growth and proliferation with minimum apoptotic cells. Little cell apoptosis was observed in the PS1 scaffolds for the initial 3 days study (Figure 7b), compared to PS2 (Figure 8b). However, after 5 days cell growth, cell–cell contact was observed in both PS1 and PS2, with continuous monolayer formation in the latter scaffold (Figure 8e). Rhodamine-DAPI staining revealed the nuclear and actin filaments in both PS1 and PS2. Fewer actin filaments were observed in PS1 fibers for 3 days (Figure 7c) but with progression the filaments denoted astral arrangement after 5 days (Figure 7f). PS2 initially revealed cluster like arrangement of actin filaments (Figure 8c) but gradually formed thick monolayer of aligned actin filaments with nuclear infiltration after 5 days (Figure 8f). Since, SF was predicted to be at the core of the nanofibers, its concentration might not directly affect the cell growth and proliferation. However, increased SF imparted moderate hydrophilicity to PS2 scaffolds that might promote cell adherence and proliferation to a greater extent.

## 4. Conclusions

Core-shell nanofibers were electrospun efficiently with emulsion combinations of PCL-SF. The concentration of SF in the emulsion dominated the nanofiber arrangement, diameter distribution, and thickness of the core-shell of individual nanofibers. The thickness of the core being changing with SF concentration; it could be concluded that SF eventually formed the core of the nanofibers with PCL in the shell. With increase in the SF concentration, a gradual change of hydrophobicity to moderate hydrophilicity was observed in the spun nanofibers. The tensile properties of the nanofibers depicted appreciable elastic modulus and ultimate tensile strength necessary for various tissue engineering applications. The hydrophilicity and presence of SF in the core-shell nanofibers governed the adherence, migration, and expansion of cells with negligible cell apoptosis. Thus, the nanofibers could be a prospective vehicle for drug-delivery, growth factors, or biomolecule encapsulation in future tissue engineering applications.

## Figures and Tables

**Table 1:** Properties of the different emulsion combinations.

Emulsion Combination	Nanofiber Name	Spinnability	Nanofiber Diameter
PCL 10%-SF 0%	PS0	Y	539.8 ± 93.2 nm
PCL 10%-SF 10%-70:30	PS1	Y	336.3 ± 60.4 nm
PCL 10%-SF 10%-50:50	PS2	Y ^¥^	448.8 ± 198.0 nm
PCL 10%-SF 10%-30:70	PS3	N	1021.6 ± 351.9 nm
PCL 10%-SF 5%-70:30	PS4	Y	212.6 ± 43.4 nm
PCL 10%-SF 5%-50:50	PS5	Y	370.1 ± 111.43 nm

Y—Yes; N—Not spinnable; ^¥^—Spannable with secondary jets of nanofibers, random fiber.

**Table 2:** Physicomechanical properties of polymer emulsion and electrospun nanofibers.

Sample	Contact Angle (^o^)	Conductivity (µS/cm)	Viscosity at Shear Rate of 54 s^−1^ (Pas)	Elastic Modulus (MPa)	Ultimate Tensile Strength (MPa)	Maximum Strain (%)	Mean Roughness (µm)
PS0	127.0 ± 0.2	---	11.77	8.93 ± 0.76	4.8 ± 0.05	58.5 ± 0.71	4.52
PS1	110.1 ± 0.6	2.104 ± 0.16	2.81	12.73 ± 0.68	4.7 ± 0.16	62.7 ± 1.36	4.98
PS2	63.6 ± 1.3	2.711 ± 0.22	0.35	32.00 ± 0.51	3.7 ± 0.34	54.1 ± 0.86	6.24
PS4	121.7 ± 1.6	11.453 ± 0.61	0.40	16.74 ± 0.52	4.9 ± 0.18	64.3 ± 1.61	4.71
PS5	94.5 ± 0.4	21.783 ± 1.53	0.30	25.61 ± 0.78	1.3 ± 0.14	40.4 ± 0.73	5.52

## Supplementary Materials

The following are available online at http://www.mdpi.com/2306-5354/5/3/68/s1, Figure S1: 3D surface roughness contour of (a) PS0, (b) PS1, (c) PS2, (d) PS4, (e) PS5, and (f) tabular representation of mean surface roughness of the electrospun scaffolds.
