# Supplementary material for: Core-Shell Nanofibrous Scaffold Based on Polycaprolactone-Silk Fibroin Emulsion Electrospinning for Tissue Engineering Applications
Source: Bioengineering (Basel). 2018 Aug 21;5(3):68. doi: 10.3390/bioengineering5030068 (PMC6164798; doi:10.3390/bioengineering5030068)
Supplement: Supplementary file 1 [file bioengineering-05-00068-s001.pdf]

bioengineering

MDPI

Supplementary Material

The surface roughness of the electrospun mats were analyzed under a 3D surface profilometer (Make—Bruker, Germany, Model—ContourGT-K (AUTOMATED)). It was observed that with incorporation of SF in the nanofiber mats the surface roughness increased. The increase in the concentration of SF affected the orientation of nanofiber deposition leading to increased surface roughness (Figure S1).

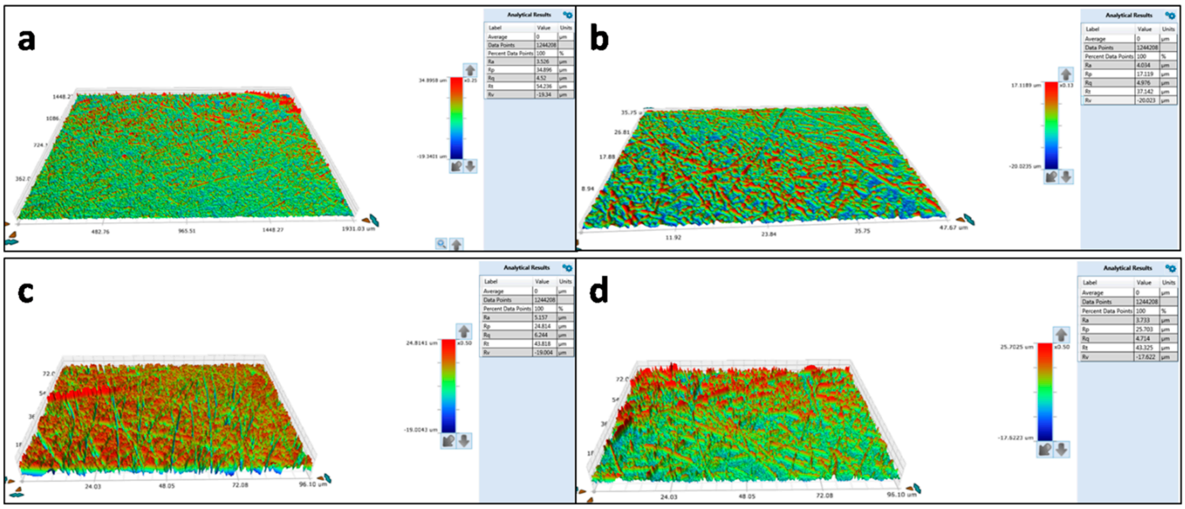

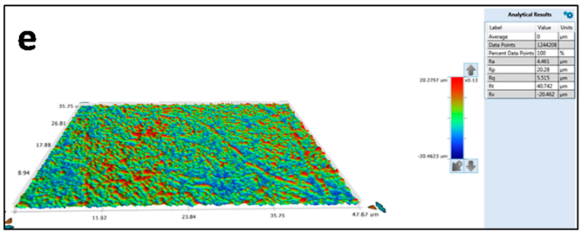

f

Scaffolds	Mean roughness (µm)
PS0	4.52
PS1	4.98
PS2	6.24
PS4	4.71
PS5	5.52

Figure S1. 3D surface roughness contour of (**a**) PS0, (**b**) PS1, (**c**) PS2, (**d**) PS4, (**e**) PS5 and (**f**) tabular representation of mean surface roughness of the electrospun scaffolds.
